# Partial Least Squares Regression Performs Well in MRI-Based Individualized Estimations

**DOI:** 10.3389/fnins.2019.01282

**Published:** 2019-11-27

**Authors:** Chen Chen, Xuyu Cao, Lixia Tian

**Affiliations:** School of Computer and Information Technology, Beijing Jiaotong University, Beijing, China

**Keywords:** machine learning, multi-label learning, regression, classification, resting state fMRI, resting state functional connection, Human Connectome Project, partial correlation

## Abstract

Estimation of individuals’ cognitive, behavioral and demographic (CBD) variables based on MRI has attracted much research interest in the past decade, and effective machine learning techniques are of great importance for these estimations. Partial least squares regression (PLSR) is an attractive machine learning technique that can accommodate both single- and multi-label learning in a simple framework, while its potential for MRI-based estimations of CBD variables remains to be explored. In this study, we systemically investigated the performance of PLSR in MRI-based estimations of individuals’ CBD variables, especially its performance in simultaneous estimation of multiple CBD variables (multi-label learning). We performed the study on the dataset included in the HCP S1200 release. Resting state functional connections (RSFCs) were used as features, and a total of 10 CBD variables (e.g., age, gender, grip strength, and picture vocabulary) were estimated. The results showed that PLSR performed well in both single- and multi-label learning. In fact, the present estimations were better than those reported in literatures, as indicated by stronger correlations between the estimated and actual CBD variables, as well as high gender classification accuracy (97.8% in this study). Moreover, the RSFCs that contributed to the estimations exhibited strong correlations with the CBD variable estimated, that is, PLSR algorithm automatically selected the RSFCs closely related to one CBD variable to establish predictive models for the variable. Besides, the estimation accuracies based on RSFCs among 100, 200, and 300 regions of interest (ROIs) were higher than those based on RSFCs among 15, 25, and 50 ROIs; the estimation accuracies based on RSFCs evaluated using partial correlation were higher than those based on RSFCs evaluated using full correlation. In addition to the aforementioned virtues, PLSR is efficient in model training and testing, and it is simple and easy to use. Therefore, PLSR can be a favorable choice for future MRI-based estimations of CBD variables.

## Introduction

Individual differences in brain structure and function exist even among persons with no diagnosable neurological or psychiatric diseases. Numerous studies have been performed to relate these differences to variability in CBD variables (for reviews, see Kanai and Rees, 2011; Parasuraman and Jiang, 2012). Besides these studies on the neural basis of individual differences in CBD variables using statistical techniques, there is a surge of interest in estimating individuals’ CBD variables using machine learning techniques based on MRI-derived brain structural and functional measures (for reviews, see Arbabshirani et al., 2017; Rathore et al., 2017). These studies have taken an important step toward individualized estimations of CBD variables.

In the studies on individualized estimations of CBD variables, machine learning techniques play critical roles. A variety of machine learning techniques have been used to establish estimation models. The most frequently used techniques are support vector machine (SVR) (Feis et al., 2013; Ullman et al., 2014), elastic net (E-Net) (Tian et al., 2016; Cui and Gong, 2018), relevance vector regression (RVR) (Stonnington et al., 2010; Franke et al., 2012; Gong et al., 2014) and linear regression (Finn et al., 2015; Rosenberg et al., 2016). Each of these techniques is specialized for single-label learning; that is, the models built based on these techniques estimate one variable at a time. The extensive use of these techniques in MRI-based estimations benefit from three of their advantages: (1) being simple and easy to use; (2) offering high estimation accuracies; and (3) enabling later inferences of the biological significance underlying the estimations.

Besides the aforementioned single-label learning techniques, multi-label learning techniques have attracted widespread attention in the region of machine learning in recent years. For MRI-based estimations, multi-label learning enables simultaneously estimation of multiple CBD variables and thus can provide richer information as compared to single-label learning. For instance, for the case of the diagnosis of Alzheimer’s disease (AD), multi-label learning enables simultaneous estimation of categorical variable (with value of either ‘AD’ or healthy control) and numerical variables such as Mini Mental State Examination (MMSE) and Alzheimer’s Disease Assessment Scale-Cognitive Subscale (ADAS-Cog) (Zhang et al., 2012), while single-label learning can only estimate one variable at a time. Moreover, multi-label learning is expected to obtain equally high, or even higher, estimation accuracies by utilizing the correlation information among different labels (for a review, see Zhang and Zhou, 2014). To date, there have been several studies on MRI-based estimations of CBD variables using multi-label learning techniques (Zhang et al., 2012; Wan et al., 2014; Yu et al., 2016; Adeli et al., 2019). However, the complexity of the multi-label learning frameworks in these studies hampers their widely use in the region, even though relatively high estimation accuracies can be obtained. Moreover, complicated learning frameworks make it difficult to infer the biological significance underlying the estimations. In fact, effective, simple and convenient multi-label learning techniques for MRI-based estimations of CBD variables are lacking.

Partial least squares regression (PLSR) is a machine learning technique that can solve both single- and multi-label learning problems. Partial least squares models relationships between sets of observed variables with “latent variables” (Wold, 1982). By virtue of its computational efficiency (projecting 1000s of features into a very low-dimensional subspace), as well as its ability of achieving dimensionality reduction and model learning simultaneously, PLSR can be a valuable choice for prediction purpose. In fact, PLSR has been reported to perform well in such areas as computer vision (Guo and Mu, 2011), food science (Cozzolino et al., 2005), remote sensing (Hansen and Schjoerring, 2003), and geoinformation (Cho et al., 2007). Especially, it was reported to perform well in simultaneously estimating individuals’ age and classifying their gender and ethnicity based on face images (Guo and Mu, 2011, 2013). In the neuroimaging region, Krishnan et al. (2011) foresaw the potential of PLSR in MRI-based estimations and described its main computational steps with a small artificial example. Afterward, PLSR has been used to estimate individuals’ full scale IQ (Yang et al., 2013), motor skill acquisition (Wu et al., 2014), episodic memory performance (Meskaldji et al., 2016), long-term-memory scores (Meskaldji et al., 2016), clinical depression scores (Yoshida et al., 2017), attentional abilities (Yoo et al., 2018), gender (Zhang et al., 2018), and future processing speed (Kuceyeski et al., 2018) based on MRI data. To note, a single variable was estimated in most of these studies. That is, the potential of PLSR for MRI-based estimations of CBD variables remains to be explored, especially its potential for simultaneous estimations of individuals’ multiple CBD variables.

In addition to machine learning techniques, appropriate brain structural and functional measures (features) are also important for MRI-based estimations. RSFCs have been one of the most commonly used features in MRI-based estimations of CBD variables (for a review, see Rathore et al., 2017). RSFCs measure the synchrony of resting state fMRI signals between brain regions and have been suggested to reflect the intrinsic architecture of the human brain (Biswal et al., 1995; Fox and Raichle, 2007). With the widespread availability of resting-state fMRI datasets of large sample sizes, RSFC has become one of the few most frequently used features for MRI-based estimations. To date, RSFCs have been reported to be effective for estimating a variety of CBD variables, such as sustained attention (Rosenberg et al., 2016; Yoo et al., 2018), intelligence quotient (Finn et al., 2015), creativity (Beaty et al., 2018), visual/verbal memory (Siegel et al., 2016), and temperament traits (Jiang et al., 2018), as well as age (Dosenbach et al., 2010) and gender (Feis et al., 2013; Zhang et al., 2018). These studies demonstrated the effectiveness of RSFCs for estimations of CBD variables.

In this study, we systemically investigated the performance of PLSR in MRI-based estimations of individuals’ CBD variables (sometimes referred to as “labels” below), especially its performance in multi-label learning. We performed the study on the large sample resting state fMRI data from the HCP S1200 release. The RSFCs among the ROIs defined by ICA were used as features, and four sets of estimations were performed to make a full understanding of the performance of PLSR in MRI-based estimations. The first set was performed to test the performance of PLSR on MRI-based multi-label learning. Here, we systemically analyzed the influences of ROI definition, RSFC evaluation strategies and the number of latent variables upon the estimations. In the second set, we simultaneously estimated another group of labels that have been estimated in other studies (Cui and Gong, 2018^1^), to provide an intuitive idea about the relative effectiveness of PLSR in MRI-based estimations. The third set was to test whether PLSR can accommodate more variables, by entering all CBD variables included in the aforementioned two groups into a single estimation model. The fourth set tested the performance of PLSR on single-label learning.

## Materials and Methods

### Dataset

The publicly available dataset HCP S1200 release^2^ was used in this study. For the current study, HCP data have two major advantages. First, the high quality of HCP data guarantees the reliability of RSFCs and CBD variables (Feinberg et al., 2010; Moeller et al., 2010; Setsompop et al., 2012; Xu et al., 2012), which are the basis for later PLSR model training. Second, the sample size of HCP S1200 is large enough to avoid any possible overfitting (Cui and Gong, 2018), which is often the case in estimations of CBD variables based on small sample MRI data.

The HCP S1200 release includes high quality multi-modal neuroimaging, behavioral and genotype data of nearly 1,100 healthy young adults (Van Essen et al., 2013; Glasser et al., 2016). Resting state fMRI data and several CBD variables were analyzed in this study. The following is a detailed description of the data we used.

Four resting state fMRI runs were acquired over 2 days for each subject. Each run lasted 15 min, with an isometric spatial resolution of 2 mm and a temporal resolution of 0.7 s. Details about data acquisition could be found in Smith et al. (2013). Based on rigorous quality control, the resting state fMRI data of 1,003 subjects were made available.

A total of 10 CBD variables were used in this study, and details about the variables can be found in Table 1. We used age, education, composite scores of fluid cognition (CSFC), crystallized cognition (CSCC), and overall cognition (CSOC) as the main estimation variables, and this group of variables will sometimes be referred to as “main labels” below. We chose to estimate age and intelligence for the consideration that they play important roles in human life. In fact, a number of studies have been performed on the estimations of age and intelligence based on MRI (Dosenbach et al., 2010; Finn et al., 2015). Age and education level were measured in years, and CSFC, CSCC, and CSOC were obtained based on the NIH Cognition Battery Toolbox. As age was also included as a label here, non-age-adjusted CSFC, CSCC, and CSOC (raw scores) were used in this study. The CSFC was designed to measure individuals’ abilities to adapt to novel situations in everyday life, such as solving problems, thinking and acting quickly, and encoding new episodic memories. The CSCC was designed to measure the accumulated store of verbal knowledge and skills in individuals. The CSOC is derived from the CSFC and CSCC, and measures the overall intelligence level of an individual (see Akshoomoff et al., 2013 for more details about the three variables). Within the 1,003 subjects whose fMRI data were available, 13 subjects with missing labels were excluded. Thus, 990 subjects were included in the main analyses of this study, and their HCP IDs are provided in Supplementary Table S1.

**TABLE 1:** Cognitive, behavioral and demographic variable information.

**Label**	**Range**	**Description**
	**(Mean ± std)**	
Age	22–37	Age of the participant in years
	(28.721 ± 3.702)	
Education	11–17	Years of education completed:
	(14.956 ± 1.773)	11- = 11; 12; 13; 14; 15; 16; 17 + = 17
Cognition score of	86.680–145.170	Measures individuals’ abilities of adapting to novel situations in everyday life;
fluid composite	(115.616 ± 11.500)	Evaluated using the NIH Cognition Battery Toolbox.
Cognition score of	90.950–153.950	Measures accumulated store of verbal knowledge and skills in individuals.
crystallized composite	(118.053 ± 9.866)	Evaluated using the NIH Cognition Battery Toolbox.
Cognition score of	88.950–153.360	Measures the overall intelligence level of an individual.
total composite	(122.552 ± 14.454)	Evaluated using the NIH Cognition Battery Toolbox.
Gender^∗^	F(0): 523/M(1): 463	Gender of the participant
Reading recognition^∗^	84.200–150.710	Measures the reading decoding skill.
	(117.190 ± 10.594)	Evaluated using Oral Reading Recognition Test included in the NIH Cognition Battery Toolbox.
Picture vocabulary^∗^	90.690–148.544	Measures the general vocabulary knowledge.
	(116.998 ± 9.449)	Estimated using Picture Vocabulary Test included in the NIH Cognition Battery Toolbox.
VSPLOT^∗^	1–26	Measures the abilities of spatial orientation.
	(15.015 ± 4.405)	Estimated using Variable Short Penn Line Orientation Test included in the NIH Cognition Battery Toolbox.
Gripstrength^∗^	55.290–154.010	Measures the relative force the participant was able to generate using his/her dominant hand.
	(116.782 ± 11.288)	Estimated using Grip Strength Dynamometry Test included in the NIH Cognition Battery Toolbox.

*^∗^The range and the mean (std) of the label were based on 986 subjects.*

To further provide an intuitive idea about the relative effectiveness of PLSR in MRI-based estimations, another group of CBD variables were used in this study, which will sometimes be referred to as “Supplementary labels” below. This group of variables includes gender, grip strength, reading recognition, picture vocabulary and VSPLOT, and raw scores (rather than age-adjusted scores) of these variables were used in this study. Cui and Gong (2018) previously estimated the latter four variables using six single-label learning methods, and we estimated these four variables here to provide an intuitive idea about the performance of PLSR. Gender was also included for the consideration that the estimation of individuals’ gender is a typical classification problem. That is, it is convenient to test whether PLSR can solve classification and regression problems simultaneously by including gender as an additional variable. Four subjects with missing labels were further excluded in this analysis, and data of 986 subjects were analyzed. The HCP IDs of the four subjects further excluded here are also provided in Supplementary Table S1.

### fMRI Data Pre-processing and RSFC Analyses

Resting state functional connections provided on the HCP website^2^ were directly used as features in the current study, and no standardization or scaling was performed on the RSFCs before entering them into the PLSR-based estimation models. Before RSFC calculation, the resting state fMRI data of each subject underwent spatial and temporal pre-processing. The MRI data pre-processing pipelines of HCP were primarily built using tools from FSL (Jenkinson et al., 2012) and FreeSurfer (Fischl, 2012; Glasser et al., 2013).

Spatial pre-processing was designed to remove spatial artifacts from the data without removing other potentially useful information (Glasser et al., 2013). The spatial pre-processing steps include spatial distortion correction, head motion correction, B0 distortion correction, spatial registration to the T1w structural images and finally to the standard MNI template, resampling to 2 mm, global intensity normalization, and masking out non-brain voxels. More details about spatial pre-processing could be found in Glasser et al. (2013).

Temporal pre-processing was designed to eliminate artifacts and noise, while preserving neuro-biologically relevant fluctuations as much as possible (Smith et al., 2013). The temporal pre-processing steps include slow drift removal by weak high-pass temporal filtering, identification of artifactual components using FSL FIX, removal of artifacts and head motion based on linear regression. More details about temporal pre-processing could be found in Smith et al. (2013).

Regions of interest time-series were then extracted from the pre-processed resting state fMRI images based on ICA. Specifically, Group-ICA was first applied to the pre-processed resting state fMRI images at six dimensionalities (*d* = 15, 25, 50, 100, 200, 300). The time-series corresponding to the components for each subject were then estimated by multiple spatial regression of his/her pre-processed resting state fMRI image against the group-ICA spatial maps. The “components” will be referred to as “ROIs” for consistency with tradition. According to Smith et al. (2013), ICA-based ROI definition may provide “a more ‘accurate’ reflection of the connectivity structures in the data,” may guarantee later network modeling “not to be rank deficient,” and may “identify remaining artifactual process in the data.” Later RSFC analyses were based on the time-series obtained above, which will be referred to as ROI time-series below.

The HCP website provided 12 variations of RSFCs, each of which was evaluated using one of six ROI definitions (15, 25, 50, 100, 200, and 300 ROIs) and one of two connectivity definitions (full correlation and partial correlation). Unlike full correlation, which is sensitive to both direct and indirect connections, partial correlation can theoretically provide a better approximation to direct connections (Marrelec et al., 2006; Smith et al., 2013). The partial-correlation-based RSFCs were evaluated using FSLNets^3^, with *method* set to ridge regression, and *rho* set to 0.01^4^. Empirically, we performed the study based mainly on RSFCs among 200 ROIs evaluated using partial correlation, and the influences of ROI definitions and RSFC evaluation strategies were also analyzed (see section “Estimations Based on PLSR”).

### Estimations Based on PLSR

Partial least squares model the relationships between two sets of variables by projecting them into a low-dimensional subspace of latent variables (Wold, 1982; Guo and Mu, 2011; Krishnan et al., 2011). Let X_n×N_ denote the feature matrix, where n is the number of samples, and N is the number of features, and let Y_n×M_ denote the label matrix, where M is the number of labels; then PLS decomposes X and Y into the following form:

$${\text{X}}_{n\times N}=T_{n\times d}\,{\left(P_{N\times d}\right)}^{\text{T}}+E_{n\times N}$$

(1) $$Y_{n\times M}=U_{n\times d}\,{\left(Q_{M\times d}\right)}^{T}+F_{n\times M}$$

where T and U are matrices of the *d* extracted score vectors (latent variables), P and Q represent matrices of loadings, and E and F are the residual errors. Partial least squares decompose X and Y to obtain the maximized covariance between T and U. Based on X, Y, U, T, an explicit *N*×*M* matrix B that satisfies the following linear relationship can be obtained:

(2) $$Y_{n\times M}=X_{n\times N}\,B_{N\times M}+{\text{F}}_{n\times M}^{*}$$

This linear relationship enables us to estimate the labels (here, the CBD variables) of unseen subjects based on their features (here, the RSFCs). PLSR was performed in this study using the *plsregress* function in MATLAB R2017b. There is only one hyper-parameter for PLSR algorithm, and it is the number of latent variables (*d*-value in Eq. 1). In this study, *d*-value was empirically set to 50, and its influences on PLSR-based estimations were also analyzed.

**FIGURE 1:**
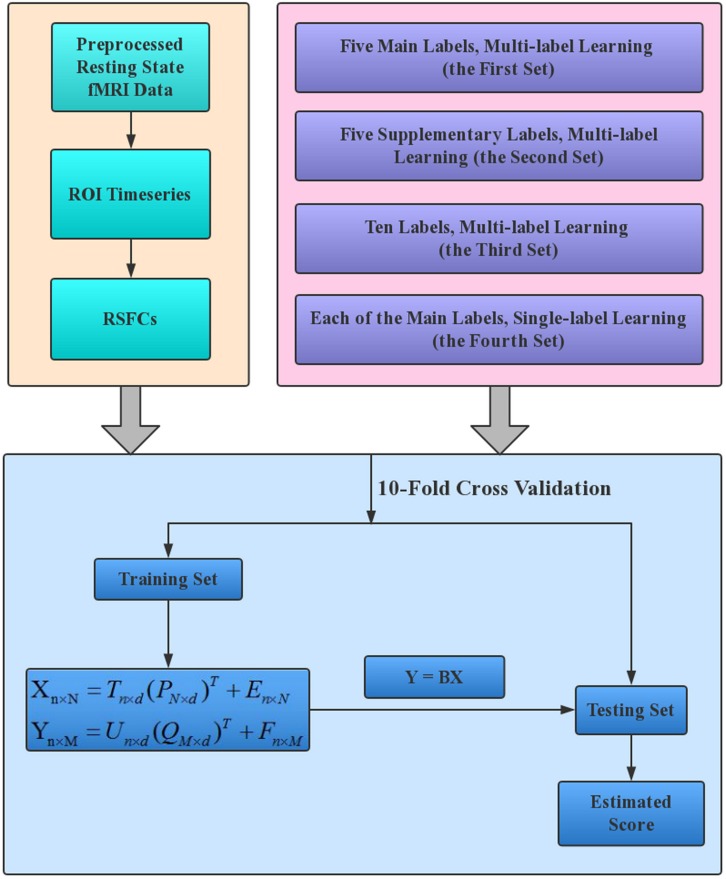
The working flowchart of the proposed estimation framework. The resting state fMRI data of HCP 1200S release were analyzed in this study, and RSFCs provided on the HCP website (https://db.humanconnectome.org/) were directly used to establish the estimation models. Each of the four label sets in the upper right box corresponds to one set of estimations.

A schematic overview of our estimation framework is shown in Figure 1. Four sets of estimations were performed to make a full understanding of the performance of PLSR in MRI-based estimations. The four sets were different only in the labels that were entered into the estimation model. Specifically, in the first set of estimations, all five main labels were entered into the model to evaluate the performance of PLSR on MRI-based multi-label learning; in the second set, we simultaneously estimated the five Supplementary labels. This set is expected to provide an intuitive idea about the relative effectiveness of PLSR in MRI-based estimations, as these labels have formerly been estimated using other machine learning techniques (e.g., SVR, elastic net) based on HCP resting state fMRI data (Cui and Gong, 2018^1^). In the third set, all 10 CBD variables were estimated simultaneously to test whether PLSR can accommodate more variables. In the fourth set, each of the five main labels was estimated separately, to evaluate the performance of PLSR on MRI-based single-label learning. For gender classification in the second and third sets, we set the label for male/female as 1/0, and the estimated gender was thresholded at 0.5 to make the final decision (≥0.5 was classified as male, and < 0.5 was classified as female).

In this study, three factors may influence the estimations based on PLSR, and these are the number of latent variables (*d*-value in Eq. 1), the ROI definition (15, 25, 50, 100, 200, and 300 ROIs) and RSFC evaluation strategies (full correlation and partial correlation). To test the influence of each of the three factors, further analyses were performed for the first set of estimations by fixing the other two factors to change the remaining one. First, the five main labels were simultaneously estimated with the number of latent variables (*d*-value) changed from 10 to 150 in steps of 10, based on partial correlation among 200 ROIs, to test the influence of the number of latent variables. Second, the five main labels were simultaneously estimated based on partial correlation among each of the other five sets of ROIs (15, 25, 50, 100, and 300 ROIs), with *d* = 50 for PLSR, to test the influence of ROI definition. Finally, the five main labels were simultaneously estimated based on full correlation among 200 ROIs, with *d* = 50 for PLSR, to test the influence of RSFC evaluation strategies.

A 10-fold cross-validation strategy was implemented to evaluate the performance of the PLSR. Specifically, all subjects were randomly divided into 10 subsets. In each loop of the 10-fold cross validation, one subset (99 subjects) was used as the testing set, and the other 9 subsets (891 subjects) were used as the training set. The estimation model was constructed (obtaining matrix B in Eq. 2) based on all training samples and then used to estimate the CBD variables of all testing samples. The training and testing procedures were repeated 10 times so that each of the 10 subsets was used as the testing set once.

The estimation performance was calculated with the Pearson correlation coefficient (*R*-value) between the actual and the estimated CBD variable and the RMSE between them. Permutation analysis was performed to test the significance of the *R*-values by randomly shuffling the CBD variables 5,000 times and repeating the estimation process. As the permutation analyses were time consuming, we performed permutation analyses only on the first and fourth sets of estimations. The *P*-values of the empirical correlation values, based on their corresponding null distributions, were computed as follows:

(3) $$P=\frac{1+N_{\mathrm{StrongerCorrelations}}}{1+N}$$

where *N* is the number of permutations (here, *N* = 5,000) and N_*StrongerCorrelations*_ is the number of stronger correlations between the estimated and permuted CBD variable (as compared to that based on the non-permuted CBD variable).

### Evaluating the Contribution of RSFCs

Based on Eq. 2, a linear relationship between the RSFCs and CBD variables can be established. This linear relationship may facilitate our evaluation of the contribution of the RSFCs to the estimations. In this study, as cross-validation was used to evaluate the performance of PLSR, slightly different linear models (as indicated by matrix B in Eq. 2) were built for each of the 10 loops. We averaged the 10 B matrices to obtain an average weight matrix ($\bar{B}$), and the contribution of the *i*th RSFC to the estimation of the *j* th CBD variable was evaluated as ${\bar{B}}_{\text{ij}}$. The significance of ${\bar{B}}_{\text{ij}}$ was again computed based on the aforementioned 5,000 permutations as follows:

(4) $$P=\frac{1+N_{L\,\arg e\,r\,A\,b\,s\,o\,l\,u\,t\,e\,{\bar{B}}_{i\,j}}}{1+N}$$

where ${\text{N}}_{L\,\arg e\,r\,A\,b\,s\,o\,l\,u\,t\,e\,{\bar{B}}_{i\,j}}$is the number of larger absolute ${\bar{B}}_{i\,j}$ in the 5000 permutations, as compared to that based on the non-permuted CBD variable. RSFCs whose weights satisfy *P* < 0.05 were regarded as making significant contributions to the estimation of a CBD variable.

We checked to what extent the RSFCs made significant contributions in multi-label learning overlapped those with significant weights for single-label learning (according to the *P*-values based on 5,000 permutations). Through this analysis, we meant to investigate whether the RSFCs would change if a few more CBD variables were entered into the PLSR model.

To investigate whether the RSFCs that made significant contributions to the estimation of a CBD variable were of biological significance, we directly correlated each RSFC with the CBD variable. Furthermore, we evaluated the contribution of the RSFCs from a network perspective. Specifically, we first clustered the ROIs into 10 functional networks based on their RSFCs using affinity propagation algorithm. The contribution of each network was then evaluated by summing up the contribution of all ROIs within it, and the contribution of each ROI was evaluated by the number of RSFCs (made significant contribution) associated with the ROI. We also evaluated the contribution of inter-network connections by the number of RSFCs (made significant contribution) between each pair of network.

## Results

### Performances of PLSR in Multi- and Single-Label Learning

**FIGURE 2:**
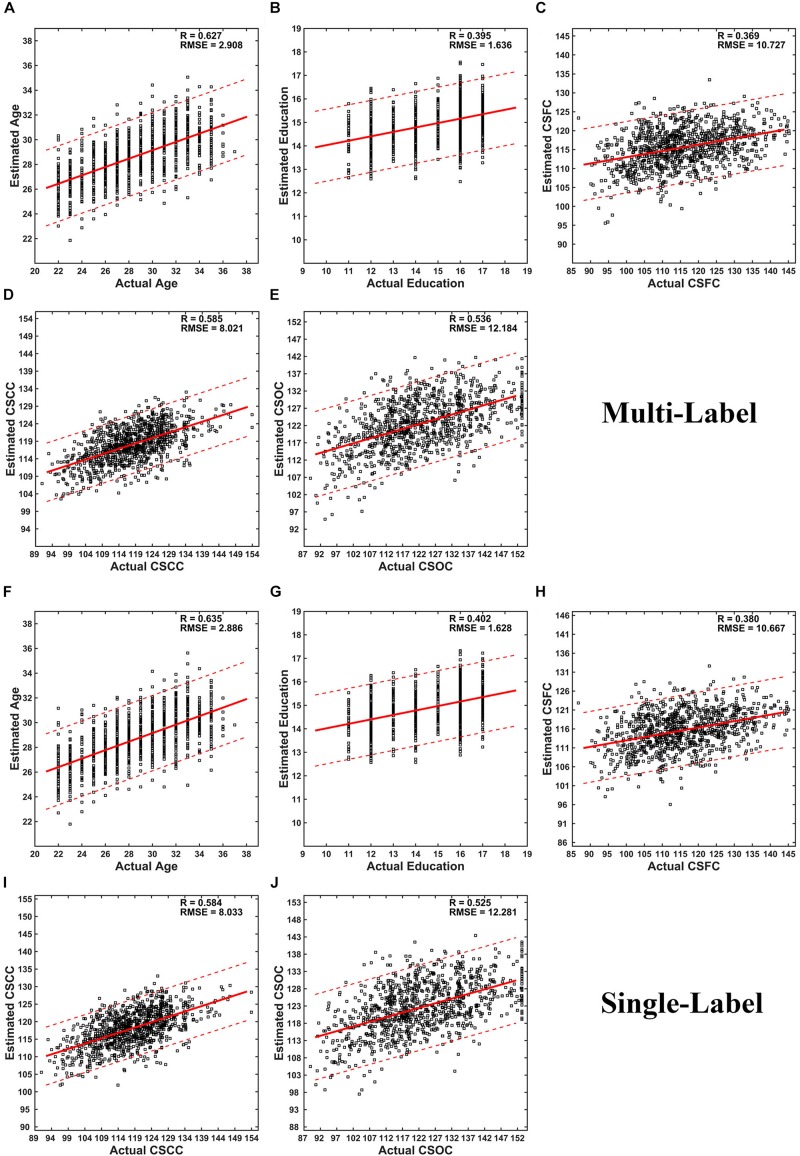
Scatter plots of estimated vs. actual labels. **(A–E)** were based on the first set of estimations (multi-label learning), and **(F–J)** were based on the fourth set of estimations (single label learning). **(A,F)** Age; **(B,G)** Education; **(C,H)** CSFC; **(D,I)** CSCC; **(E,J)** CSOC.

**FIGURE 3:**
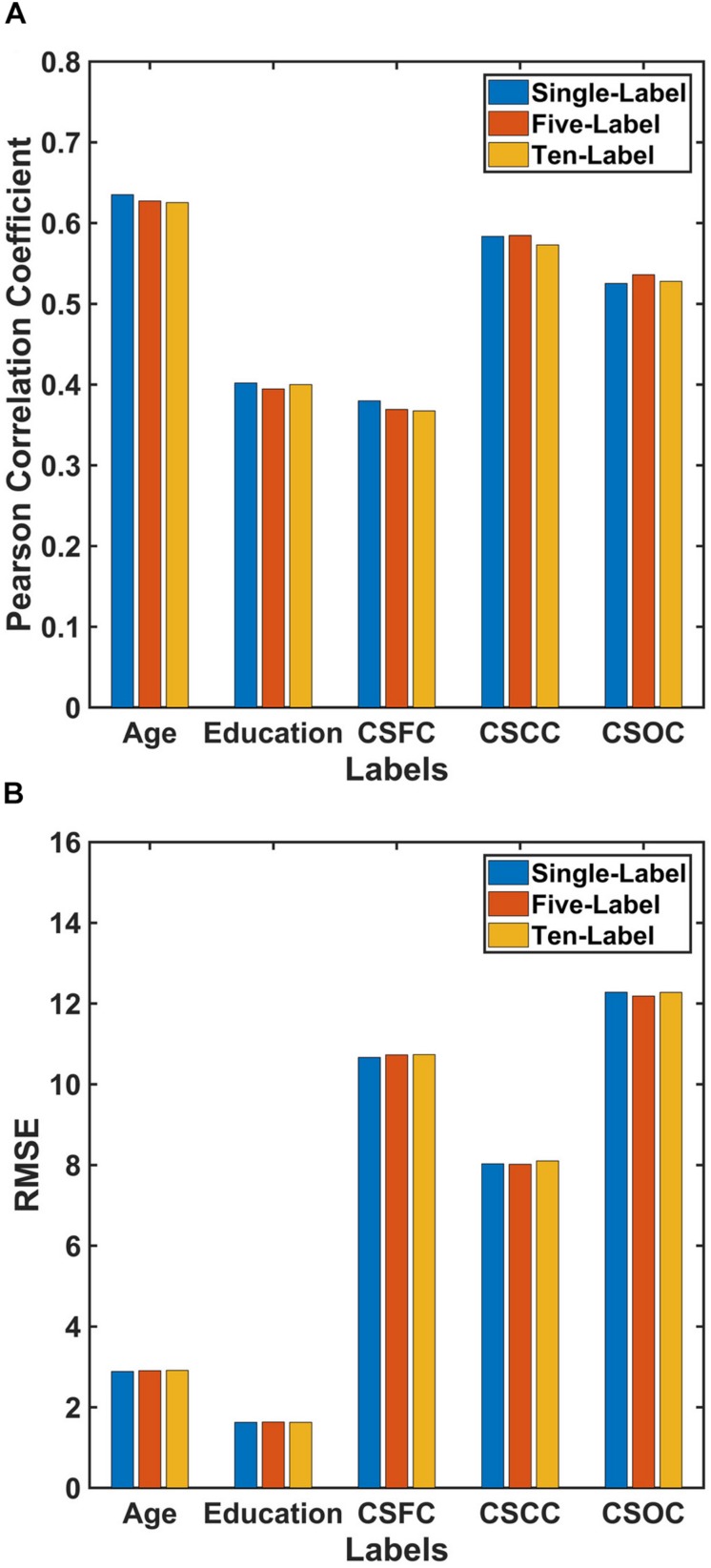
Comparison of the performances of PLSR for single- and multi-label learning. The reported values were **(A)** Pearson correlation coefficient and **(B)** RMSE. The performances of the single-label PLSR were comparable to those of the multi-label PLSR, and adding additional five labels into the model had limited effect upon the estimation accuracies.

Partial least squares regression performed well in MRI-based estimations for both single- and multi-label learning purpose (Figures 2, 3 and Tables 2, 3). For simultaneous estimation of the main labels (the first set of estimations), *R*-values of 0.627, 0.395, 0.369, 0.585, and 0.536 were obtained for age, education, CSFC, CSCC, and CSOC, respectively (Figures 2A–E). For each of the five variables, no stronger correlation was observed in the 5,000 permutations. That is, each of the five *R*-values corresponded to a *P*-value of 0.0002. In fact, the largest *R*-values in the 5,000 permutations were by far smaller than those based on actual CBD variables, which were 0.157, 0.146, 0.161, 0.142, and 0.161 for age, education, CSFC, CSCC, and CSOC, respectively.

**TABLE 2:** Performances of PLSR in the four sets of estimations.

**The first set: multi-label learning, five main labels**					

	**Age**	**Education**	**CSFC**	**CSCC**	**CSOC**
*R*	0.627	0.395	0.369	0.585	0.536
RMSE	2.908	1.636	10.727	8.021	12.184

**The second set: multi-label learning, five Supplementary labels**					

	**Grip strength**	**Reading recognition**	**Picture vocabulary**	**VSPLOT**	**Gender^∗^**

*R*	0.701	0.522	0.555	0.376	97.6%(ACC)
RMSE	8.066	9.038	7.871	4.109	0.996(AUC)

**The third set: multi-label learning, ten labels**					

	**Age**	**Education**	**CSFC**	**CSCC**	**CSOC**

*R*	0.625	0.400	0.367	0.573	0.528
RMSE	2.914	1.629	10.738	8.101	12.278

	**Grip strength**	**Reading recognition**	**Picture vocabulary**	**VSPLOT**	**Gender^∗^**

*R*	0.704	0.519	0.546	0.382	97.8%(ACC)
RMSE	8.033	9.059	7.920	4.093	0.996(AUC)

**The fourth set: single-label learning, five main labels**					

	**Age**	**Education**	**CSFC**	**CSCC**	**CSOC**

*R*	0.635	0.402	0.380	0.584	0.525
RMSE	2.886	1.628	10.667	8.033	12.281

*^∗^For gender classification, the classification accuracy (ACC) and the area under curve (AUC) were provided.*

**TABLE 3:** Comparison of the estimations based on PLSR in this study to those based on elastic net listed on the HCP website^∗^.

		**Education**	**Grip strength**	**Reading recognition**	**Picture vocabulary**	**VSPLOT**
**PLSR**	***R***	0.400	0.704	0.519	0.546	0.382
	**CoD**	0.156	0.494	0.269	0.297	0.136
**Elastic net**	***R***	0.28	0.65	0.16	0.27	0.25
	**CoD**	–0.05	0.34	–0.19	–0.18	–0.01

*^∗^https://db.humanconnectome.org/megatrawl/3T_HCP820_MSMAll_d200_ts2/megatrawl_1/. The estimation accuracies of the other five CBD variables (age, gender, CSFC, CSCC, and CSOC) were not available on the HCP website. Our results were based on the third set of estimations. The estimations based on PLSR in this study were better than those based on elastic net as listed on the HCP website. CoD stands for “coefficient of determination,” which has been used to evaluate the performance of the predictive models together with the *R*-value on the HCP website. CoD is evaluated as follows: CoD = 1 – Variance of Estimation Error/Variance of the Variable, and higher CoD values indicate better estimations.*

The results regarding the simultaneous estimation of the five Supplementary CBD variables (the second set of estimations) are listed in Table 2. A gender classification accuracy of 97.6%, together with *R*-values of 0.701, 0.522, 0.555, and 0.376 for the estimations of grip strength, reading recognition, picture vocabulary and VSPLOT were obtained.

Table 2 also provided the results regarding the simultaneous estimations of 10 CBD variables (the third set of estimations). It can be seen that including five additional CBD variables into the model did not influence the estimations of the main labels. For instance, the *R*-value for age estimation changed from 0.627 to 0.625 here, and the gender classification accuracy changed from 97.6 to 97.8% here.

The current estimation accuracies of grip strength, reading recognition, picture vocabulary, and VSPLOT (*R* = 0.704, 0.519, 0.546, 0.382, respectively) were higher than those reported in Cui and Gong (2018) (not more than 0.55, 0.35, 0.35, 0.25, respectively), in which six commonly used machine learning algorithms were utilized. The estimation accuracies in this study were also higher than those listed on the HCP website^5^, which were based on the same RSFCs as were used in this study but obtained using elastic net, and Table 3 is a direct comparison of our results and those listed on the HCP website. In fact, when we estimated the five main labels using three widely used single-label learning techniques, namely, SVR, E-Net and RVR, based on RSFCs among 200 ROIs evaluated using partial correlation, the estimation accuracies were much lower than those based on based on PLSR. For instance, the correlation between the estimated and actual ages were *R* = 0.413, 0.392, 0.405 for SVR, E-Net, and RVR, respectively, as compared to *R* = 0.627 for PLSR (for more details, please see Supplementary Table S4).

The performance of PLSR in MRI-based single-label learning (of the five main labels, the fourth set of estimations) can be found in Figures 2F–J, 3 and Table 2. There were only subtle differences between the accuracies of single- and multi-label learning. For instance, the estimation of age was slightly better based on single-label learning (*R* = 0.635, compared to *R* = 0.627 for multi-label learning), while the estimation of the CSOC was slightly better based on multi-label learning (*R* = 0.536, compared to *R* = 0.525 for single-label learning).

**FIGURE 4:**
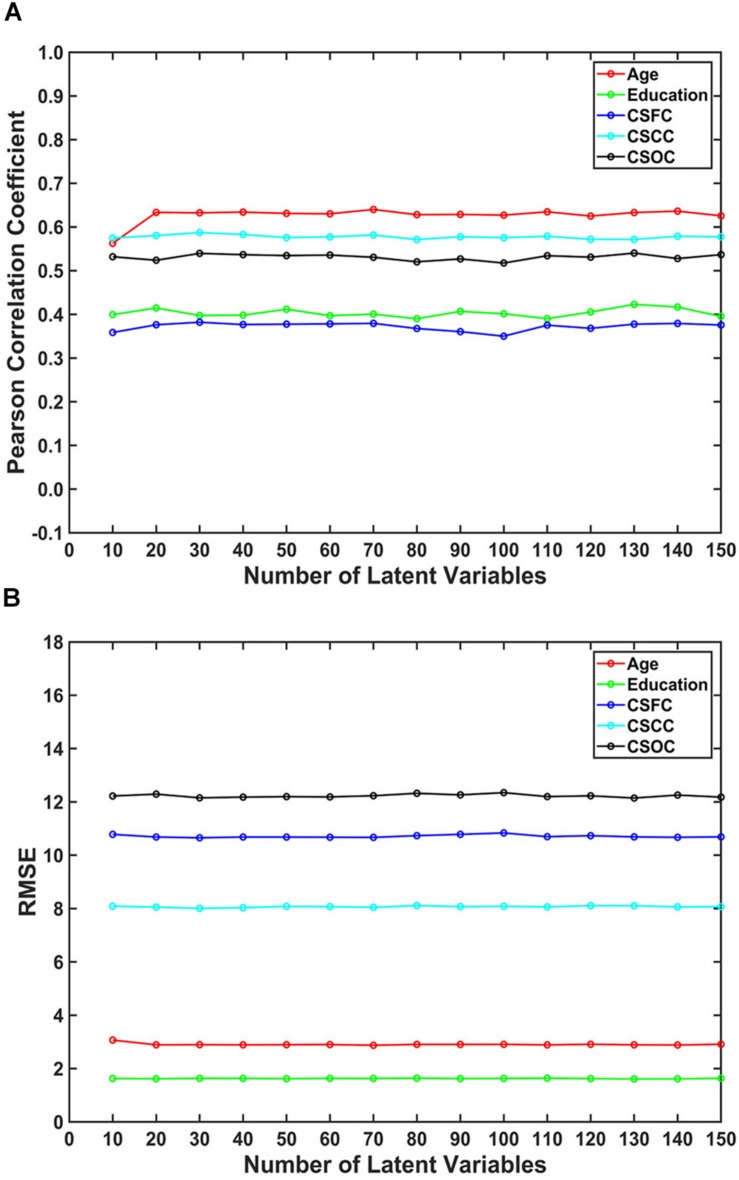
The influence of the number of latent variables on the estimations. The reported values were **(A)** Pearson correlation coefficient and **(B)** RMSE. The number of latent variables ranged from 10 to 150. The results indicated that multi-label PLSR was relatively stable when the number of latent variables varied over a wide range (here, 10 ∼ 150).

The number of latent variables (*d*-value) is an important factor for PLSR. On analyzing its influence, it was found that *d*-value had a limited effect on the estimations (Figure 4). Specifically, only subtle changes of the *R*-value and RMSE were observed, with *d*-values ranging from 10 to 150. This result indicated that PLSR was relatively robust to *d*-value selection.

### Influences of ROI Definition and RSFC Evaluation Strategies

**FIGURE 5:**
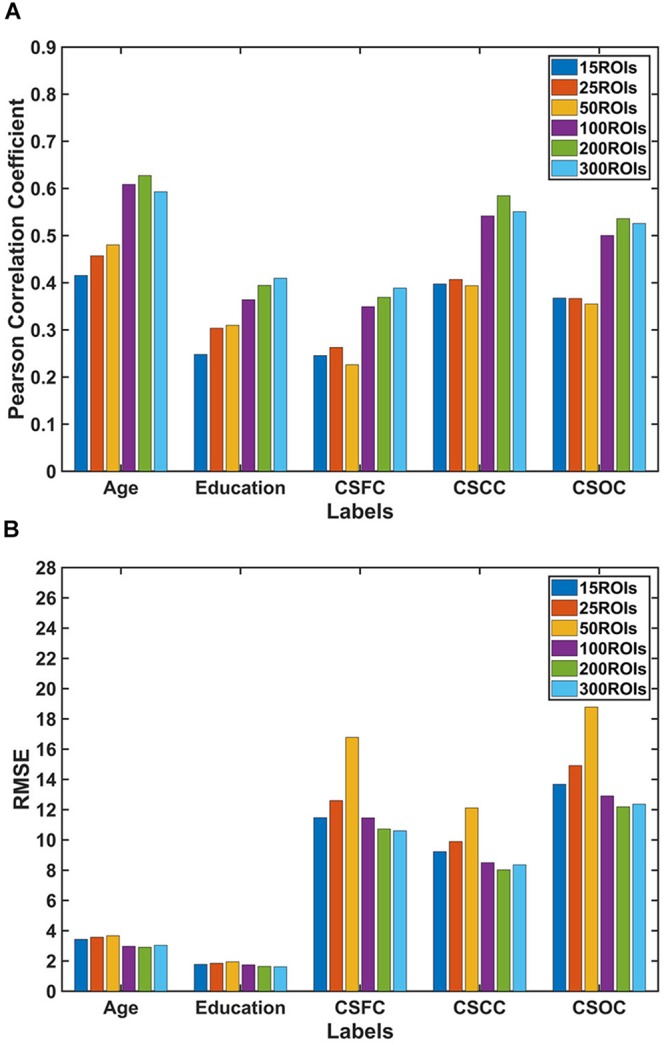
Estimations based on different ROI definition strategies. The reported values were **(A)** Pearson correlation coefficient and **(B)** RMSE. The results indicated that estimations based on 100, 200, and 300 ROIs were better than those based on 15, 25, and 50 ROIs.

**FIGURE 6:**
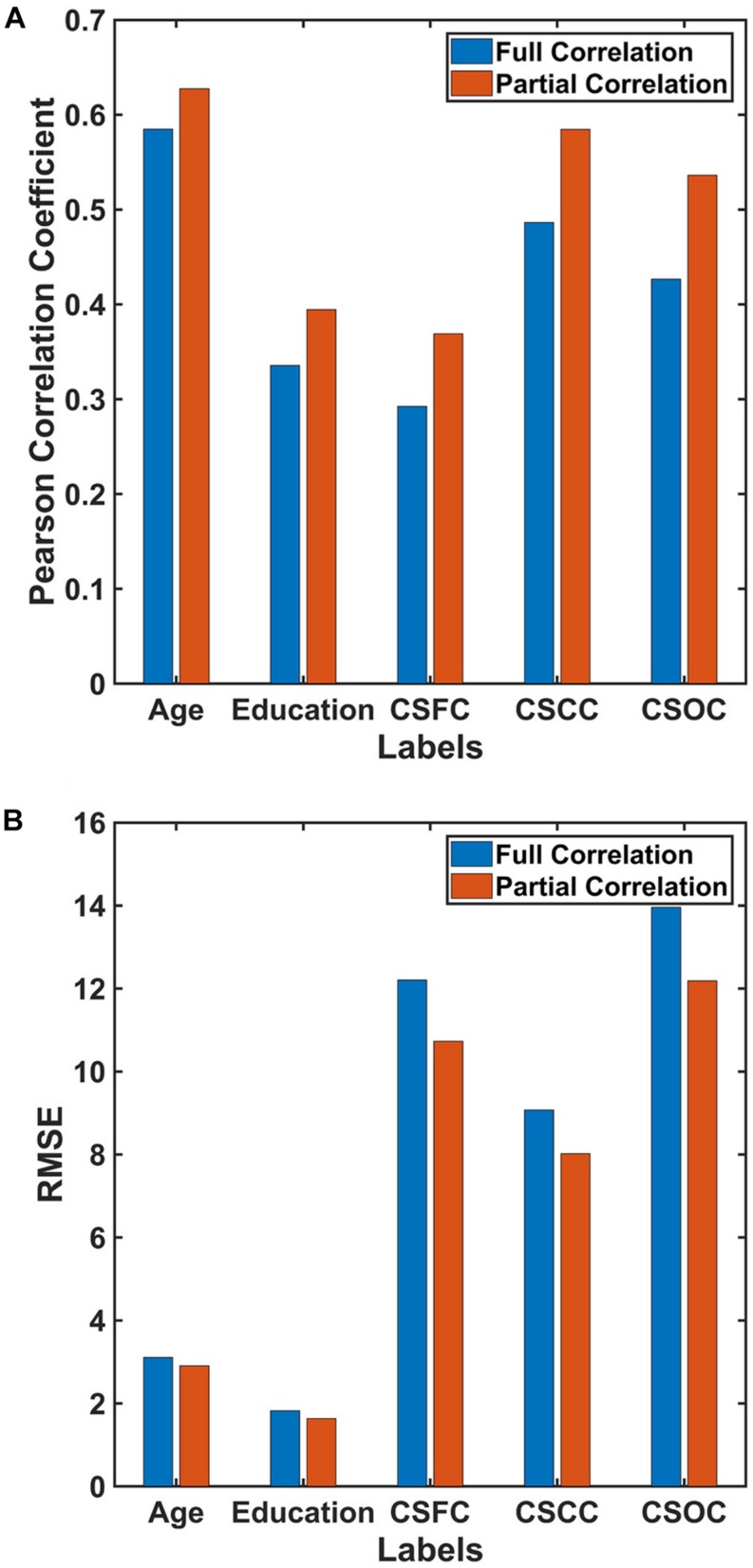
Estimations based on different RSFC evaluation strategies. The reported values were **(A)** Pearson correlation coefficient and **(B)** RMSE. The results indicated that estimations based on RSFCs evaluated using partial correlation were better than those based on RSFCs evaluated using full correlation.

For the first set of estimations (multi-label learning of the five main labels), we further evaluated the influences of ROI definition and RSFC evaluation strategies. Figure 5 illustrates the influence of ROI definition strategy on the estimations. The estimation accuracies based on 100, 200, and 300 ROIs were relatively higher than those based on 15, 25, and 50 ROIs. A comparison of the estimations based on RSFCs evaluated using full correlation and partial correlation can be found in Figure 6. Obviously, partial-correlation-based RSFCs generally outperformed full-correlation-based RSFCs.

### RSFCs Made Significant Contributions to Estimations

**FIGURE 7:**
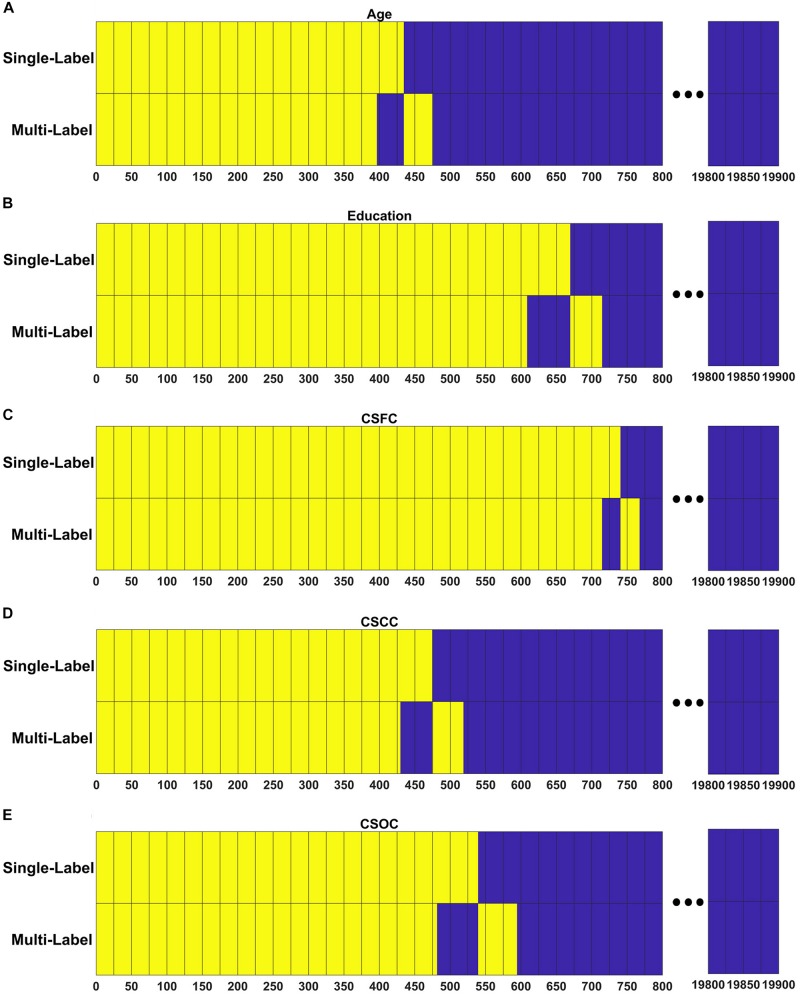
Resting state functional connections made significant contributions shared across the PLSR models established in the first and fourth set of estimations. For each model, RSFCs made significant contributions are marked with yellow, RSFCs made non-significant contributions are marked with blue, and common features between the two models are vertically aligned. Respectively, among the 437, 653, 741, 473, and 536 RSFCs that made significant contributions to the simultaneous estimations of **(A)** age, **(B)** education, **(C)** CSFC, **(D)** CSCC, and **(E)** CSOC, 396, 608, 714, 429, and 481 were in common between the multi- and single-label learning.

Figure 7 demonstrates the extent to which the RSFCs with significant weights in the multi-label learning overlapped those with significant weights for the single-label learning. A large percentage of the RSFCs contributed to multi- and single-label estimations were common. For instance, among the 437 RSFCs with significant weights in the multi-label estimation of age, 396 RSFCs had significant weights in the single-label estimation (Figure 7A).

**FIGURE 8:**
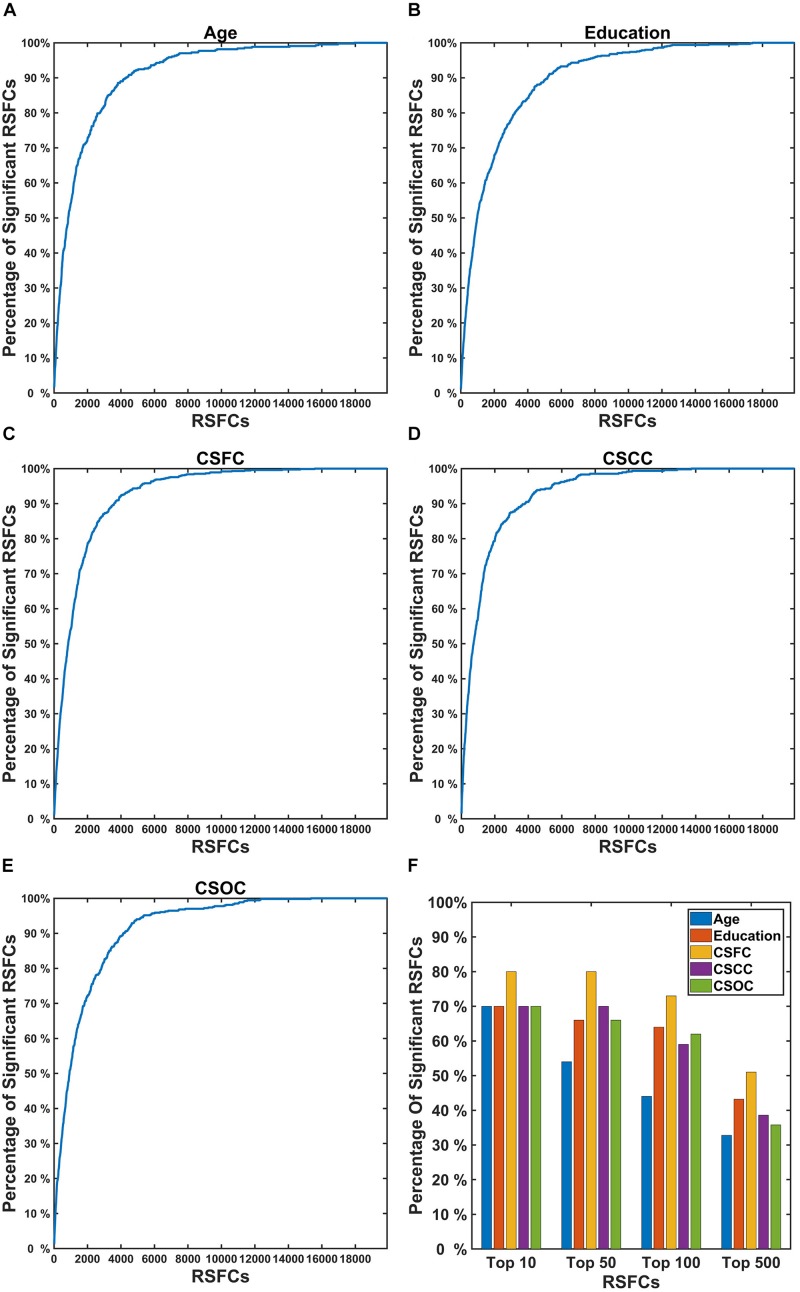
Percentage of RSFCs that made significant contributions to the estimations of a CBD variable among the RSFCs that strongly correlated with that variable. In the first five subplots **(A–E)**, the X coordinate indicates the sequence number of the correlation (absolute value, sorted in descending order) between the RSFCs and the CBD variable [**(A)** for Age, **(B)** for Education, **(C)** for CSFC, **(D)** for CSCC, and **(E)** for CSOC]; the Y coordinate indicates the percentage of RSFCs that made significant contributions to that variable (among all RSFCs that made significant contributions to that variable). **(F)** Represents the percentage of RSFCs that made significant contributions to the estimations among the top 10, 50, 100, and 500 RSFCs that were most correlated with the CBD variable. The results were obtained based on the first set of estimations. A large percentage of the RSFCs that contributed to the estimation of a CBD variable were observed to have strong correlation with that variable.

Figure 8 shows the percentage of RSFCs that made significant contributions to the estimations among the RSFCs that strongly correlated with the variable. It can be seen that quite a few RSFCs that made significant contributions to the estimation of a CBD variable had a strong correlation with that variable. For instance, among the 10 RSFCs that showed the strongest correlation with age, seven were observed to make a significant contribution to the estimation of age (Figure 8F). These strong correlations indicate that the RSFCs made significant contributions to estimations were of biological significance.

**FIGURE 9:**
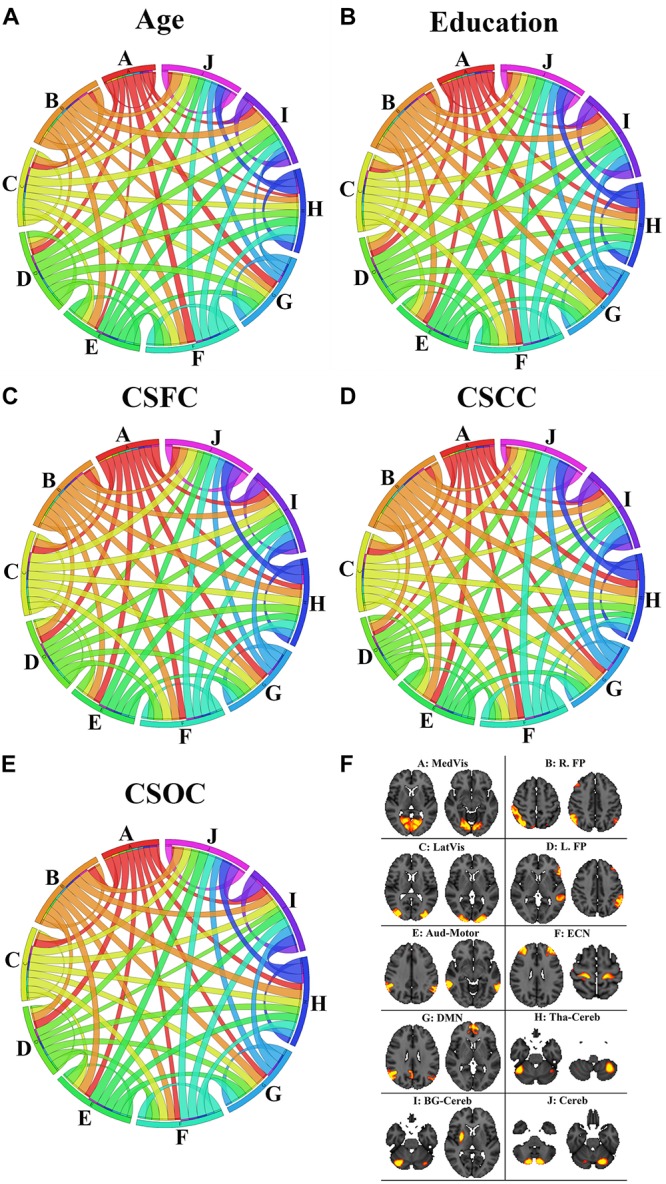
The contribution of the RSFCs to the estimation of age **(A)**, education **(B)**, CSFC **(C)**, CSCC **(D)**, and CSOC **(E)**. The circular diagram indicates the relative contribution of the functional networks, with each functional network indicated by one color. The networks are the medial visual network (**A**: MedVis), the right fronto-parietal network (**B**: R. FP), the lateral visual network (**C**: LatVis), the left fronto-parietal network (**D**: L. FP), the auditory motor network (**E**: Aud-Mot), the executive control network (**F**: ECN), the default mode network (**G**: DMN), the thalamus cerebellum network (**H**: Tha-Cereb), the basal ganglia cerebellum network (**I**: BG-Cereb) and the cerebellum (**J**: Cereb). Two typical spatial maps of each network can be found in subfigure **(F)**. Each ribbon links two functional networks, and the ribbon size scales with the contribution of the RSFCs between the networks it linked.

Figure 9 illustrates the contribution of the RSFCs from the perspective of functional networks. According to Figure 9, the network contribution was slightly different when estimating different variables. For instance, the medial visual network contributed relatively less in the estimation of age (Figure 9A), as compared to the estimation of other variables (Figures 9B–E). The inter-network connections that contributed to the estimations of the five main labels were also slightly different. For instance, the RSFCs between the medial and lateral visual networks contributed relatively less to the estimation of education, as compared to the estimation of CSOC.

## Discussion

It is valuable to estimate individuals’ CBD variables based on neuroimaging data, as these estimations may eventually lead to a better understanding of the neural basis that gives rise to individual differences in these variables, and may potentially assist in the clinical diagnosis of neuropsychiatric diseases. Machine learning techniques play critical roles in these estimations. Krishnan et al. (2011) foresaw the potential of PLSR in MRI-based estimations. Afterward, quite a few studies have been performed on MRI-based estimations using PLSR, but a majority of these studies estimated one CBD variable at a time. That is, the potential of PLSR for MRI-based estimations of CBD variables remains to be explored, especially its potential for multi-label learning. In this study, we systemically investigated the performance of PLSR in MRI-based estimations of individuals’ CBD variables. The following is a detailed discussion of the results.

### PLSR Performed Well in MRI-Based Estimations

In the current study, PLSR was observed to perform well in simultaneous estimations of individuals’ multiple CBD variables based on resting state fMRI (Figures 2, 3 and Tables 2, 3). According to the Pearson correlations between the estimated and actual CBD variables (*R*-values, each corresponding to a *P* = 0.0002 in this study), the present estimations were better than those in two other studies based on the HCP resting state fMRI data but using single-label learning techniques (Cui and Gong, 2018) (see footnote 5). Specifically, the *R*-values for grip strength, reading recognition, picture vocabulary and VSPLOT obtained in this study (0.704, 0.519, 0.546, 0.382) was uniformly higher than those reported by Cui and Gong (2018) (not more than 0.55, 0.35, 0.35, 0.25), in which the four variables were estimated with six commonly used machine learning regression algorithms. The relatively higher estimation accuracy in the current study support the effectiveness of PLSR in MRI-based estimations, though better ROI definition and RSFC evaluation strategies (as will be discussed below) may also contribute to better estimations in this study. In fact, based on the same RSFC set (among 200 ROIs, and estimated using partial correlation), the current estimations were better than those listed on the HCP website, which was based on elastic net (Table 3). Moreover, when we estimated the five main labels using three widely used machine learning techniques, namely, SVR, E-Net and RVR, based on RSFCs among 200 ROIs evaluated using partial correlation, the estimation accuracies based on PLSR were uniformly higher than those based on the three techniques.

In addition to its relatively high estimation accuracy, PLSR exhibited four advantages in MRI-based estimations in this study. First, PLSR can solve both single- and multi-label learning problems. PLSR has been reported to perform well in estimating a variety of CBD variables (Yang et al., 2013; Wu et al., 2014; Meskaldji et al., 2016; Yoshida et al., 2017; Kuceyeski et al., 2018; Yoo et al., 2018; Zhang et al., 2018) based on MRI data. To note, a single variable was estimated in most of the studies. The current results indicate that, in addition to single label learning, PLSR can perform comparably well when multiple CBD variables were simultaneously estimated (Figures 2, 3 and Table 2).

Second, PLSR can solve regression and classification problems simultaneously. In this study, an accuracy of 97.8% was obtained for gender classification, with the other nine CBD variables estimated simultaneously (Table 2). The present accuracy was slightly higher than that reported by Feis et al. (2013), and much higher than that reported by Zhang et al. (2018). Specifically, Zhang et al. (2018) reported a gender classification accuracy of 87% based on the resting state fMRI data of the HCP dataset. In the study by Feis et al. (2013), an accuracy of 96% was obtained based on MR images (T1-, T2-, and diffusion-weighted) using a linear support vector machine. The relatively higher gender classification accuracy in our study indicates that including the other nine CBD variables into the model may be helpful for gender classification in this study. In many cases, both continuous (e.g., MMSE score) and discrete (e.g., whether or not a subject had psychiatric disease) CBD variables are available (Zhang et al., 2012; Yoshida et al., 2017). The ability of solving classification and regression problems simultaneously enables PLSR to provide richer information and higher estimation accuracy in those cases.

Third, the estimations were relatively stable when the number of latent variables (*d*-value) changed over a wide range (Figure 4). This indicates that PLSR is not sensitive to the choice of *d*-value. It should be noted that the selection of *d*-values is not unlimited. In fact, when the *d*-value was set to larger than 200, the estimations deteriorate dramatically (Supplementary Figure S1). The reason for this deterioration is not known, and further studies are expected to address the issue.

Finally, PLSR is efficient in model training and testing, and it is simple and easy to use. PLSR is very fast in learning, and even faster in testing, capable of quickly reducing the original high-dimensional data into low dimensions. In this study, it took only 1.3729 s to reduce the original 19,900-dimensional RSFCs (based on 200 ROIs) into 50-dimensional latent variables on a PC with a 3.00 GHz Intel(R) Core(TM) i5-8500 CPU processor. Once the partial least squares decomposition is completed, the subsequent testing process involves only the linear product of matrices, which is even faster. A fast testing process is beneficial for practical applications of PLSR.

### ROI Definition and RSFC Evaluation Strategies Had Obvious Influences Upon the Estimations

As has been mentioned, in addition to the advantages of PLSR, the relatively high estimation accuracies in this study may be partly due to better ROI definition and RSFC evaluation strategies. Proper ROI definition is critical for later RSFC evaluation, as a hidden hypothesis in the current study is that the ROIs for all subjects are same. This requirement of “same” ROI definition necessitates high-quality spatial normalization if a template were used. ICA itself can figure out subject specific ROIs that are more functionally “same” (Smith et al., 2013). The ICA-based ROI definition may be one reason for better estimations in this study, as compared to those in the study by Cui and Gong (2018), in which ROIs were defined based on the human brainnetome atlas.

Compared to estimations based on 15, 25 and 50 ROIs, the estimations based on 100, 200, and 300 ROIs were much better (Figure 5). This finding is consistent with that reported by Finn et al. (2015), in which the accuracy of individual identification based on 68 ROIs was much lower as compared to that based on 268 ROIs. Finn et al. (2015) suggest that “a relatively high-resolution parcelation contributes to the detection of individual variability and boosts identification rate.” According to Yoshida et al. (2017), estimations of clinical scores deteriorate dramatically when the standard AAL template was further subdivided into 600 ROIs. It is still unknown whether more ROIs (e.g., 600 or 1000) would impair the estimations based on PLSR. Further studies are needed to address this issue.

Estimations of all five CBD variables based on RSFCs evaluated using partial correlation were better than those based on RSFCs evaluated using full correlation (Figure 6). Partial correlation has been suggested to be a better approximation to direct connections in theory, while full correlation is more sensitive to both direct and indirect connections (Marrelec et al., 2006; Smith et al., 2013). If this were the case, the current results indicate that, by excluding the effects of indirect connections, the RSFCs evaluated based on partial correlation include less noise, and this is favorable for CBD variable estimations.

### RSFCs Made Significant Contributions to the Estimations Were of Biological Significance

Though all RSFCs were utilized to obtain the latent variables and finally to establish the linear relationship as given in Eq. 2, only a few RSFCs were observed to make significant contributions (Figure 7). Moreover, in the current study, the RSFCs that made significant contributions to multi-label estimations largely overlapped with those in single-label estimations (Figure 7). For instance, 396 of the 437 RSFCs that made significant contributions to age estimation based on the multi-label learning model were also found to make significant contributions based on a single-label learning model (Figure 7A). This result indicated that the RSFC sets utilized by PLSR were quite similar for single- and multi-label learning. We suggest that PLSR can automatically find out the RSFCs of biological significance for one CBD variable (e.g., age), irrespective of the influences of other CBD variables that were simultaneously estimated (e.g., education).

To investigate whether the RSFCs made significant contributions to the estimation of a CBD variable were of biological significance, we compared these RSFCs to those exhibited significant correlations with the variable. It was found that a majority of the RSFCs that made significant contributions to the estimation of a CBD variable also had strong correlation with the variable (Figure 8). For instance, nearly 90% of the RSFCs that made significant contributions to the estimation of age were among 4,000 (among 19,900) RSFCs that showed the strongest correlation with age (Figure 8A). This indicated that the estimations based on PLSR were largely dependent upon RSFCs of biological significance.

When the ROIs were clustered into 10 functional networks, each network contributed differently to the estimation of the five main labels (Figure 9). As has been mentioned, the medial visual network made relatively less contribution to the estimation of age (Figure 9A), as compared to the estimation of other variables (Figures 9B–E). The medial visual network is thought to be important for preliminary visual information processing. The present finding is consistent with the suggestion by Dosenbach et al. (2010), which indicates that the networks responsible for preliminary sensory functions mature early and aging late. That is, medial visual network may be relatively stable during early adulthood, so it contribute less to age estimation in this study (as subjects included in this study aged 22∼37 years). Inter-network connections were also observed to make slightly different contribution to the estimation of the five main labels (Figure 9). One example is that the connection between the medial and lateral visual networks contributed relatively less to the estimation of education (as compared to CSOC). The medial visual network plays an important role in preliminary visual information processing, and lateral visual network is critical for high-order visual information processing. the current finding may be consistent with the common sense that individuals’ ability of visual information processing is less dependent on education, but the speed and quality of visual information processing (supported by the medial and lateral visual networks) may exert some influence upon individuals’ cognitive abilities (as evaluated by CSOC).

### Other Methodological Issues

Two methodological issues should be addressed. First, family structure was not considered in this study. Most subjects in this study had at least one blood relative, and many of them were twins (Van Essen et al., 2013). The homogeneity of the sample may make the estimation accuracies too optimistic, as many families will be split across training and testing sets. To avoid over-optimistic estimation accuracies, we further performed 10-fold cross validation with family structure taken into account, by ensuring that no family was split across training and testing sets. The results indicated that whether or not considering family structure has limited influence on the final accuracies (Supplementary Table S2).

Another methodological issue is that the kernel trick was not considered in this study. The kernel trick has been widely used in the area of machine learning to capture the non-linear relationships between features and labels. According to Guo and Mu (2011), kernel PLSR resulted in a smaller error compared to linear PLSR. When we simultaneously estimated the five main labels using kernel PLSR, with the RBF function used as kernel and σ empirically set to 150, the estimation accuracies increased slightly (Supplementary Table S3). This result indicated that kernel PLSR can be a favorable choice for future MRI-based estimations.

## Conclusion

In summary, we systemically investigated the performance of PLSR in MRI-based estimations of individuals’ CBD variables. We found that PLSR performed well in both simultaneous estimation of multiple CBD variables and estimation of a single CBD variable. Furthermore, our study demonstrated five advantages of PLSR in MRI-based estimations, which are attractive to researchers in the field. First, PLSR can solve both single- and multi-label learning problems. Second, PLSR can solve regression and classification problems simultaneously. Third, the PLSR algorithm is relatively robust to the number of latent variables. Then, PLSR enables later inferences of the biological significance underlying the estimations. Finally, PLSR is efficient in model training and testing, and it is simple and easy to use. Besides, the choice of ROI definition and RSFC evaluation strategies are also critical for the estimations. Specifically, our results indicated that RSFCs evaluated using partial correlation had obvious advantages over those evaluated using full correlation, and the estimations based on RSFCs among 100, 200, and 300 ROIs were much better than those based on RSFCs among 15, 25, and 50 ROIs. This study used RSFCs as a test case, and it is obvious that PLSR can be easily extended to estimations based on other features (e.g., VBM evaluated based on MRI). Furthermore, PLSR is simple in principle and easy to use, so it can be widely used in future MRI-based estimations of CBD variables.

## Data Availability Statement

Publicly available datasets were analyzed in this study. This data can be found here: https://db.humanconnectome.org/.

## Ethics Statement

The studies involving human participants were reviewed and approved by NIH. The patients/participants provided their written informed consent to participate in this study.

## Author Contributions

LT and CC designed the study and analyzed the data. CC, XC, and LT wrote the manuscript and approved the submitted version.

## Conflict of Interest

The authors declare that the research was conducted in the absence of any commercial or financial relationships that could be construed as a potential conflict of interest.

## Footnotes

1 https://db.humanconnectome.org/megatrawl/3T_HCP820_MSMAll_d200_ts2/megatrawl_1/

2 https://db.humanconnectome.org/

3 https://fsl.fmrib.ox.ac.uk/fsl/fslwiki/FSLNets

4 https://db.humanconnectome.org/megatrawl/HCP820_MegaTrawl_April2016.pdf

5 https://db.humanconnectome.org/megatrawl/

## Abbreviations

CBD: cognitive, behavioral and demographic
CSCC: composite score of crystallized cognition
CSFC: composite score of fluid cognition
CSOC: composite score of overall cognition
E-Net: elastic net
ICA: independent component analysis
PLSR: partial least squares regression
RMSE: root mean square error
ROI: regions of interest
RSFCs: resting state functional connections
RVR: relevance vector regression
SVR: support vector regression.
