# Supplementary material for: Partial Least Squares Regression Performs Well in MRI-Based Individualized Estimations
Source: Front Neurosci. 2019 Nov 27;13:1282. doi: 10.3389/fnins.2019.01282 (PMC6890557; doi:10.3389/fnins.2019.01282)
Supplement: Supplementary file 1 [file Table_1.docx]

**Supplementary Materials**


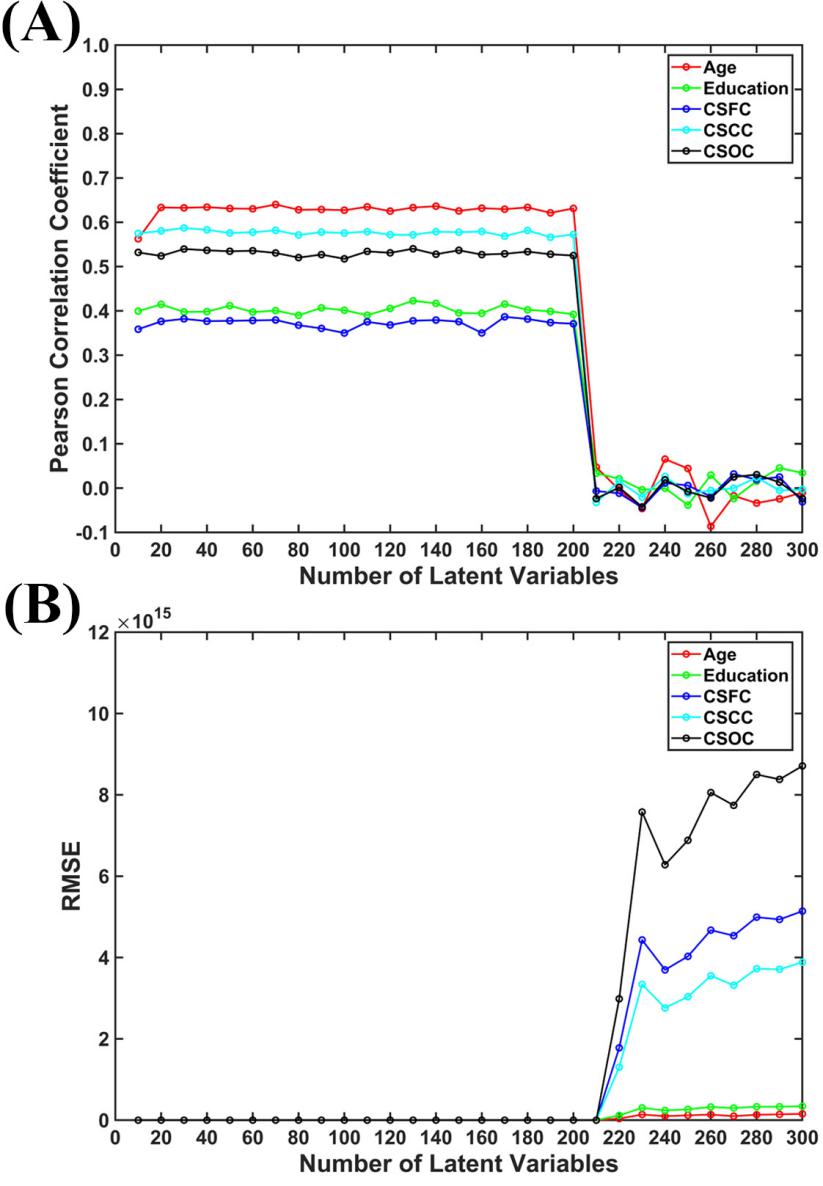


**Fig. S1. The influence of the number of latent variables (ranged from 10 to 300).** The reported values were (A) Pearson correlation coefficient and (B) RMSE. The results indicated that performance of multi-label PLSR deteriorate dramatically once the number of latent variables were too large (here, larger than 200).

**Table S1. All HCP IDs of included subjects in HCP S1200 dataset (990 in total).**

| 100206 | 100307 | 100408 | 100610 | 101006 | 101107 | 101309 | 101915 | 102008 | 102109 | 102311 | 102513 |
| --- | --- | --- | --- | --- | --- | --- | --- | --- | --- | --- | --- |
| 102614 | 102715 | 102816 | 103010 | 103111 | 103212 | 103414 | 103515 | 103818 | 104012 | 104416 | 104820 |
| 105014 | 105115 | 105216 | 105620 | 105923 | 106016 | 106319 | 106521 | 106824 | 107018 | 107321 | 107422 |
| 107725 | 108020 | 108121 | 108222 | 108323 | 108525 | 108828 | 109123 | 109325 | 110007 | 110411 | 110613 |
| 111211 | 111312 | 111413 | 111716 | 112112 | 112314 | 112516 | 112920 | 113215 | 113316 | 113619 | 113922 |
| 114217 | 114318 | 114419 | 114621 | 114823 | 114924 | 115017 | 115219 | 115320 | 115724 | 115825 | 116524 |
| 116726 | 117021 | 117122 | 117324 | 117930 | 118023 | 118124 | 118225 | 118528 | 118730 | 118831 | 118932 |
| 119025 | 119126 | 120111 | 120212 | 120414 | 120515 | 120717 | 121416 | 121618 | 121921 | 122317 | 122620 |
| 122822 | 123117 | 123420 | 123521 | 123723 | 123824 | 123925 | 124220 | 124422 | 124624 | 124826 | 125222 |
| 125424 | 125525 | 126325 | 126426 | 126628 | 127226 | 127327 | 127630 | 127731 | 127832 | 127933 | 128026 |
| 128127 | 128632 | 128935 | 129028 | 129129 | 129331 | 129634 | 130013 | 130114 | 130316 | 130417 | 130518 |
| 130619 | 130720 | 130821 | 130922 | 131217 | 131419 | 131722 | 131823 | 131924 | 132017 | 132118 | 133019 |
| 133625 | 133827 | 133928 | 134021 | 134223 | 134324 | 134425 | 134627 | 134728 | 134829 | 135124 | 135225 |
| 135528 | 135629 | 135730 | 135932 | 136126 | 136227 | 136631 | 136732 | 136833 | 137027 | 137128 | 137229 |
| 137431 | 137532 | 137633 | 137936 | 138130 | 138231 | 138332 | 138534 | 138837 | 139233 | 139435 | 139637 |
| 139839 | 140117 | 140824 | 140925 | 141119 | 141422 | 141826 | 142828 | 143224 | 143325 | 143426 | 143830 |
| 144125 | 144226 | 144428 | 144731 | 144832 | 144933 | 145127 | 145632 | 145834 | 146129 | 146331 | 146432 |
| 146533 | 146735 | 146836 | 146937 | 147030 | 147636 | 147737 | 148032 | 148133 | 148335 | 148436 | 148840 |
| 148941 | 149236 | 149337 | 149539 | 149741 | 149842 | 150625 | 150726 | 150928 | 151223 | 151324 | 151425 |
| 151526 | 151627 | 151728 | 151829 | 151930 | 152225 | 152427 | 152831 | 153025 | 153126 | 153227 | 153429 |
| 153631 | 153732 | 153833 | 153934 | 154229 | 154330 | 154431 | 154532 | 154734 | 154835 | 154936 | 155635 |
| 155938 | 156031 | 156334 | 156435 | 156536 | 156637 | 157336 | 157437 | 157942 | 158035 | 158136 | 158338 |
| 158540 | 158843 | 159138 | 159239 | 159340 | 159441 | 159744 | 160123 | 160729 | 160830 | 161327 | 161630 |
| 161731 | 161832 | 162026 | 162228 | 162329 | 162733 | 162935 | 163129 | 163331 | 163432 | 163836 | 164030 |
| 164131 | 164636 | 164939 | 165032 | 165436 | 165638 | 165840 | 165941 | 166438 | 166640 | 167036 | 167238 |
| 167440 | 167743 | 168139 | 168240 | 168341 | 168745 | 168947 | 169040 | 169343 | 169444 | 169545 | 169949 |
| 170631 | 171330 | 171532 | 171633 | 172029 | 172130 | 172332 | 172433 | 172534 | 172938 | 173334 | 173435 |
| 173536 | 173637 | 173738 | 173839 | 173940 | 174437 | 174841 | 175035 | 175136 | 175237 | 175338 | 175439 |
| 175540 | 175742 | 176037 | 176239 | 176441 | 176542 | 176744 | 176845 | 177140 | 177241 | 177645 | 177746 |
| 178142 | 178243 | 178647 | 178748 | 178849 | 178950 | 179245 | 179346 | 180129 | 180230 | 180432 | 180533 |
| 180735 | 180836 | 180937 | 181131 | 181232 | 181636 | 182032 | 182436 | 182739 | 182840 | 183034 | 185038 |
| 185139 | 185341 | 185442 | 185846 | 185947 | 186040 | 186141 | 186444 | 186545 | 186848 | 187143 | 187345 |
| 187547 | 187850 | 188145 | 188347 | 188448 | 188549 | 188751 | 189349 | 189450 | 189652 | 190031 | 191235 |
| 191336 | 191437 | 191841 | 191942 | 192035 | 192136 | 192237 | 192439 | 192540 | 192641 | 192843 | 193239 |
| 193845 | 194140 | 194443 | 194645 | 194746 | 194847 | 195041 | 195445 | 195647 | 195849 | 195950 | 196144 |
| 196346 | 196750 | 197348 | 197550 | 198047 | 198249 | 198350 | 198451 | 198653 | 198855 | 199150 | 199251 |
| 199352 | 199453 | 199655 | 199958 | 200008 | 200109 | 200311 | 200513 | 200614 | 200917 | 201111 | 201414 |
| 201515 | 201818 | 202113 | 202719 | 203418 | 203923 | 204016 | 204218 | 204319 | 204420 | 204521 | 204622 |
| 205119 | 205220 | 205725 | 205826 | 206323 | 206525 | 206727 | 206828 | 206929 | 207123 | 207426 | 208024 |
| 208125 | 208226 | 208327 | 208630 | 209127 | 209228 | 209329 | 209834 | 209935 | 210011 | 210112 | 210415 |
| 210617 | 211114 | 211215 | 211316 | 211417 | 211619 | 211720 | 211821 | 211922 | 212015 | 212116 | 212217 |
| 212318 | 212419 | 212823 | 213017 | 213421 | 213522 | 214019 | 214221 | 214423 | 214524 | 214625 | 217126 |
| 217429 | 219231 | 220721 | 221319 | 223929 | 224022 | 227432 | 227533 | 228434 | 231928 | 233326 | 237334 |
| 238033 | 239136 | 239944 | 245333 | 246133 | 248339 | 249947 | 250427 | 250932 | 251833 | 255639 | 255740 |
| 256540 | 257542 | 257845 | 257946 | 263436 | 268749 | 268850 | 270332 | 274542 | 275645 | 280739 | 280941 |
| 281135 | 283543 | 285345 | 285446 | 286347 | 286650 | 287248 | 289555 | 290136 | 293748 | 295146 | 297655 |
| 298051 | 298455 | 299154 | 299760 | 300618 | 300719 | 303119 | 303624 | 304020 | 304727 | 305830 | 307127 |
| 308129 | 308331 | 309636 | 310621 | 311320 | 314225 | 316633 | 316835 | 318637 | 320826 | 321323 | 322224 |
| 325129 | 329440 | 329844 | 330324 | 333330 | 334635 | 336841 | 339847 | 341834 | 342129 | 346137 | 346945 |
| 348545 | 349244 | 350330 | 352132 | 352738 | 353740 | 356948 | 358144 | 360030 | 361234 | 361941 | 365343 |
| 366042 | 366446 | 368551 | 368753 | 371843 | 376247 | 377451 | 378756 | 378857 | 379657 | 380036 | 381038 |
| 381543 | 382242 | 385046 | 385450 | 386250 | 387959 | 389357 | 391748 | 392447 | 392750 | 393550 | 394956 |
| 395251 | 395756 | 395958 | 397154 | 397760 | 397861 | 401422 | 406432 | 406836 | 412528 | 413934 | 414229 |
| 415837 | 419239 | 421226 | 422632 | 424939 | 432332 | 433839 | 436239 | 436845 | 441939 | 445543 | 448347 |
| 449753 | 453441 | 453542 | 456346 | 459453 | 461743 | 463040 | 465852 | 467351 | 468050 | 469961 | 475855 |
| 479762 | 480141 | 481042 | 481951 | 485757 | 486759 | 495255 | 497865 | 499566 | 500222 | 506234 | 510225 |
| 510326 | 512835 | 513130 | 513736 | 516742 | 517239 | 518746 | 519647 | 519950 | 520228 | 522434 | 523032 |
| 524135 | 525541 | 529549 | 529953 | 530635 | 531536 | 531940 | 536647 | 540436 | 541640 | 541943 | 545345 |
| 547046 | 548250 | 552241 | 552544 | 553344 | 555348 | 555651 | 555954 | 557857 | 558657 | 558960 | 559053 |
| 559457 | 561242 | 561444 | 561949 | 562345 | 562446 | 565452 | 566454 | 567052 | 567759 | 567961 | 568963 |
| 570243 | 571144 | 572045 | 573249 | 573451 | 576255 | 578057 | 579665 | 579867 | 580044 | 580347 | 580650 |
| 580751 | 581349 | 581450 | 583858 | 585256 | 585862 | 586460 | 587664 | 588565 | 589567 | 590047 | 592455 |
| 597869 | 598568 | 599065 | 599469 | 599671 | 601127 | 604537 | 609143 | 611938 | 613538 | 615441 | 615744 |
| 616645 | 617748 | 618952 | 620434 | 622236 | 623844 | 626648 | 627549 | 627852 | 628248 | 633847 | 634748 |
| 638049 | 645450 | 645551 | 647858 | 654350 | 654552 | 654754 | 656253 | 656657 | 657659 | 660951 | 662551 |
| 663755 | 664757 | 665254 | 667056 | 668361 | 671855 | 672756 | 673455 | 675661 | 677766 | 677968 | 679568 |
| 679770 | 680250 | 680452 | 680957 | 683256 | 685058 | 686969 | 687163 | 690152 | 692964 | 693764 | 694362 |
| 695768 | 698168 | 700634 | 702133 | 704238 | 705341 | 706040 | 707749 | 709551 | 713239 | 715041 | 715647 |
| 715950 | 720337 | 723141 | 724446 | 725751 | 727553 | 727654 | 728454 | 729254 | 729557 | 731140 | 732243 |
| 734045 | 735148 | 737960 | 742549 | 744553 | 748258 | 748662 | 749058 | 749361 | 751348 | 753150 | 753251 |
| 756055 | 757764 | 759869 | 760551 | 761957 | 763557 | 765056 | 765864 | 767464 | 769064 | 770352 | 771354 |
| 773257 | 774663 | 779370 | 782561 | 783462 | 784565 | 788674 | 788876 | 789373 | 792564 | 792766 | 792867 |
| 793465 | 800941 | 802844 | 803240 | 804646 | 809252 | 810843 | 812746 | 814548 | 814649 | 815247 | 816653 |
| 818455 | 818859 | 820745 | 825048 | 825553 | 825654 | 826353 | 826454 | 827052 | 828862 | 832651 | 833148 |
| 833249 | 835657 | 837560 | 837964 | 841349 | 843151 | 844961 | 845458 | 849264 | 849971 | 852455 | 856766 |
| 856968 | 857263 | 859671 | 861456 | 865363 | 867468 | 869472 | 870861 | 871762 | 871964 | 872158 | 872562 |
| 873968 | 877168 | 877269 | 878776 | 878877 | 880157 | 882161 | 884064 | 885975 | 886674 | 887373 | 888678 |
| 889579 | 891667 | 894067 | 894673 | 894774 | 896778 | 896879 | 898176 | 899885 | 901038 | 901139 | 901442 |
| 902242 | 904044 | 905147 | 907656 | 908860 | 910241 | 910443 | 911849 | 912447 | 917255 | 917558 | 919966 |
| 922854 | 923755 | 926862 | 927359 | 930449 | 932554 | 933253 | 937160 | 942658 | 943862 | 947668 | 951457 |
| 952863 | 955465 | 957974 | 958976 | 959574 | 962058 | 965367 | 965771 | 966975 | 969476 | 970764 | 971160 |
| 978578 | 979984 | 983773 | 984472 | 987074 | 987983 | 989987 | 990366 | 991267 | 992673 | 992774 | 993675 |
| 994273 | 996782 | 109830* | 206222* | 236130* | 614439* |  |  |  |  |  |  |

* The subjects were further excluded in the second and third sets of estimations, as their “supplementary labels” were not available.

**Table S2. Comparison of the estimations with and without considering family structure.**

|  | | **Age** | **Education** | **CSFC** | **CSCC** | **CSOC** |
| --- | --- | --- | --- | --- | --- | --- |
| **Family Structure Considered** | **R** | 0.627 | 0.391 | 0.376 | 0.584 | 0.540 |
|  | **RMSE** | 2.909 | 1.638 | 10.688 | 8.032 | 12.155 |
| **Family Structure**  **NOT Considered** | **R** | 0.627 | 0.395 | 0.369 | 0.585 | 0.536 |
|  | **RMSE** | 2.908 | 1.636 | 10.727 | 8.021 | 12.184 |

Family structure was considered by ensuring that no family was split across training and testing sets.

**Table S3. Comparison of the estimations with and without using the kernel trick.**

|  | | **Age** | **Education** | **CSFC** | **CSCC** | **CSOC** |
| --- | --- | --- | --- | --- | --- | --- |
| **Kernel**  **PLSR** | **R** | 0.634 | 0.416 | 0.407 | 0.590 | 0.550 |
|  | **RMSE** | 2.884 | 1.616 | 10.522 | 7.986 | 12.052 |
| **Linear**  **PLSR** | **R** | 0.627 | 0.395 | 0.369 | 0.585 | 0.536 |
|  | **RMSE** | 2.908 | 1.636 | 10.727 | 8.021 | 12.184 |

RBF kernel was used, with $\sigma= 150$.

**Table S4. Comparison of the estimations based on PLSR to those based on SVR, E-Net and RVR.**

|  |  | **Age** | **Education** | **CSFC** | **CSCC** | **CSOC** |
| --- | --- | --- | --- | --- | --- | --- |
| **PLSR** | **R** | 0.627 | 0.395 | 0.369 | 0.585 | 0.536 |
|  | **RMSE** | 2.908 | 1.636 | 10.727 | 8.021 | 12.184 |
| **SVR** | **R** | 0.413 | 0.235 | 0.219 | 0.400 | 0.345 |
|  | **RMSE** | 3.618 | 1.921 | 12.164 | 9.449 | 14.252 |
| **E-net** | **R** | 0.392 | 0.269 | 0.240 | 0.397 | 0.327 |
|  | **RMSE** | 4.186 | 2.103 | 14.356 | 11.210 | 17.443 |
| **RVR** | **R** | 0.405 | 0.281 | 0.228 | 0.350 | 0.345 |
|  | **RMSE** | 3.415 | 1.715 | 11.387 | 9.320 | 13.618 |

For a fair and rigorous comparison, the five main labels (age, education, CSFC, CSCC and CSOC) were estimated using three widely used single-label learning techniques, SVR, E-Net and RVR. For SVR-based estimations, 200 RSFCs that showed the strongest correlation with the label were selected for model training (Dosenbach et al., 2010), and the optimal parameter C for each fold was selected from 16 values [2^-5^, 2^-4^, …,2^9^, 2^10^] based on inner 5-fold cross-validation (Cui et al., 2018). For E-Net-based estimations, the two parameters $\alpha$ and λ were selected from 11 values [0,0.1, …,0.9] and 16 values [2^-10^, 2^-9^, …, 2^4^, 2^5^] (Cui et al., 2018), respectively, again based on an inner 5-fold cross-validation. SVR – support vector regression; E-Net – elastic net; RVR – relevance vector regression; CSFC - composite score of fluid cognition; CSCC - composite score of crystallized cognition; CSOC - composite score of overall cognition; RMSE – root mean square error.

*Cui, Z., and Gong, G. (2018). The effect of machine learning regression algorithms and sample size on individualized behavioral prediction with functional connectivity features. Neuroimage 178, 622-637. doi:10.1016/j.neuroimage.2018.06.001*

*Dosenbach, N.U., Nardos, B., Cohen, A.L., Fair, D.A., Power, J.D., Church, J.A. et al. (2010). Prediction of individual brain maturity using fMRI. Science 329, 1358-1361. doi:10.1126/science.1194144*
